# Iguratimod as an alternative induction therapy for refractory lupus nephritis: a preliminary investigational study

**DOI:** 10.1186/s13075-020-02154-7

**Published:** 2020-03-30

**Authors:** Yuening Kang, Qingran Yan, Qiong Fu, Ran Wang, Min Dai, Fang Du, Qing Dai, Ping Ye, Chunmei Wu, Liangjing Lu, Chunde Bao

**Affiliations:** Department of Rheumatology, Renji Hospital, Shanghai Jiaotong University, School of Medicine, 145 Shandong RD, Shanghai, 200001 China

**Keywords:** Iguratimod, Refractory lupus nephritis, Induction therapy

## Abstract

**Objectives:**

Iguratimod, a novel immunomodulatory agent for rheumatoid arthritis, has been shown to be effective against murine lupus. The aim of this study was to make a preliminary evaluation of the efficacy and safety of iguratimod as salvage therapy in patients with refractory lupus nephritis (LN).

**Methods:**

We enrolled eligible patients with refractory LN, which we defined as having failed or relapsed on at least two immunosuppressant agents. After enrollment, we substituted iguratimod (25 mg twice daily) for their previous immunosuppressant agents without increasing the dose of steroids. The primary outcome was complete/partial remission (PR/CR) at week 24. Patients who achieved remission continued iguratimod as maintenance therapy over an extended follow-up.

**Results:**

The study cohort comprised 14 patients with refractory LN, 10 of whom had recent treatment failure and 4 repeated relapses with inadequate initial responses. At enrollment, none of the patients had detectable evidence of extra-renal involvement. The median prednisone dosage was 10 mg/d (IQR 0–10 mg/day). Thirteen patients were eligible for response evaluation, with one patient missed. The renal response rate was 92.3% (12/13) at week 24, with 38.5% (5/13) achieving CR and 53.8% (7/13) achieving PR. We then continued to follow up the responding patients for up to 144 weeks. Twenty-five percent of the patients (3/12) had renal relapse after initial PR. The estimated glomerular filtration rate of all patients maintained stable during follow-up. One patient had a severe adverse reaction (anemia) but recovered fully after stopping iguratimod.

**Conclusions:**

Our study supports the potential of iguratimod for treatment of refractory LN. Iguratimod could be a promising candidate drug for this condition.

## Introduction

Systemic lupus erythematosus (SLE) is an autoimmune disease that can involve multiple organs or systems [1–3]. Lupus nephritis (LN) is associated with high mortality and morbidity rates. Over recent decades, substantial progress has been made in developing immunosuppressant agents and biologic therapies [4]. However, a significant proportion of patients either do not respond to first-line immunosuppressive drugs or quickly relapse after initial remission. Approximately 10% of patients with LN will experience continued worsening of renal function and go on to develop end-stage renal disease [5].

To treat refractory LN, the European League Against Rheumatism (EULAR) recommendations suggest a switch of either cyclophosphamide (CYC) to mycophenolate mofetil (MMF) or vice versa. In addition, a switch to B cell depletion therapy with rituximab may be considered [4]. In previous studies of refractory LN, an add-on strategy has usually been adopted; for example, rituximab has been added to another immunosuppressant, typically CYC [6–8], or a calcineurin inhibitor combined with MMF [9, 10]. These strategies may help to ensure efficacy but could mask the role of the newly added drug.

In recent decades, a new immunomodulatory drug, iguratimod, has emerged as a potential candidate for the treatment of autoimmune diseases. It has been approved for treating rheumatoid arthritis (RA) in northeast Asia. According to data from RA clinical trials in Japan and China, iguratimod is superior to a placebo and non-inferior to methotrexate and sulfasalazine [11–14]. In our preclinical study on lupus, iguratimod prevented autoimmune nephritis in MRL/lpr mice, decreased the amount of proteinuria, and reduced immune complex deposition [15].

Previous studies on possible mechanisms have provided compelling evidence supporting the rationale for using iguratimod to treat lupus. Iguratimod, an immunomodulatory agent, interferes with B cell differentiation. It was found to suppress B cell production of immunoglobulins over a decade ago [16]. In a phase III clinical trial on RA, iguratimod reduced serum immunoglobulin concentrations [12, 14]. In RA and lupus animal models, iguratimod has decreased autoantibody titers, including anti-collagen antibody [17, 18] and anti-double strand (dsDNA) antibody [15]. Interestingly, iguratimod reportedly decreases peripheral plasma cell counts without affecting the total B cell population in MRL/lpr mice [15] and patients with RA who are receiving iguratimod monotherapy [19]. Further investigation has shown that iguratimod regulates the key transcription factors affecting plasma cell differentiation, especially Blimp-1, through the PKC/Egr1 axis [19]. In this study, we aimed to explore the efficacy and safety of iguratimod in patients with refractory LN.

## Methods

### Study design

This was an investigational study to assess the efficacy and safety of iguratimod in patients with refractory LN. Eligibility criteria comprised having experienced treatment failure or relapse after at least two immunosuppressant agents and had of proteinuria of no less than 1.0 g/24 h at enrollment. Failure was defined as no remission (not achieving PR or CR, see below in the “Outcomes” section) on one agent for at least 6 months. All patients gave written informed consent. The study was approved by the Ethics Committee of Renji Hospital, Shanghai, China.

### Procedures

Once the patients had been enrolled, they were prescribed oral iguratimod at a dose of 25 mg twice daily. All other immunosuppressant agents were discontinued. Meanwhile, the patients continued other medications, such as steroids, anti-malaria drugs, or angiotensin-converting enzyme/receptor inhibitor (ACEI/ARB), without dose adjustment. Details of ACEI/ARB treatment are shown in Table S1 (see Additional file 1).

Blood cell counts, liver and renal function, and 24 h urinary protein was monitored at intervals of 1–3 months. Anti-dsDNA and serum complement 3 (C3) levels were measured every 6 months or at the time of premature exit from the study.

### Outcomes

Renal complete/partial remission (PR/CR) at week 24 was used as the primary outcome. If the patients had achieved CR/PR at week 24, iguratimod was continued as maintenance therapy for long-term follow-up and evaluation.

CR was defined as a 24 h urine protein < 300 mg, normal counts of urine blood cells or casts, and normal serum creatinine, whereas PR was defined as 24 h urine protein between 300 mg and 2000 mg with at least a 50% decrease from the baseline, serum albumin concentration over 30 g/L, and serum creatinine increase no more than 25%, as described in other studies [20, 21].

Other outcomes evaluated included duration of renal response, renal flares, extra-renal flares, and safety. A renal flare was defined according to European Renal Association-European Dialysis and Transplant Association (EULAR) recommendations for the management of adult and pediatric lupus nephritis [22]. An extra-renal flare was defined as the presence of manifestations that could be attributed to SLE that required high-dose steroids.

## Results

### Characteristics of patients

From 2015 to 2018, 14 eligible patients were sequentially recruited in our center (12 women and 2 men). Ten of these patients had recent treatment failure and four had repeated inadequate responses, all four never having achieved complete remission (for patients’ history, see Additional file 2). All nephritis had been confirmed by biopsy when proteinuria was first detected (WHO class III/IV/V) [23]. Three patients agreed to a repeat biopsy before switching to iguratimod. Major clinical characteristics are shown in Table 1 and details of each patient’s previous medications are shown in Additional file 2.

**Table 1:** Demographic information, previous medications, and major outcomes for all patients

Patient	Age	Sex	Duration of LN (years)	LN class	Previous medications and outcome	Baseline TPU (g/24 h)	Baseline prednisone (mg/d)	Baseline creatine (μmol/L)	Outcome of iguratimod treatment	Follow-up time (weeks)	Follow-up
1	30	F	13	IV → III^§^	Cyc → Cs* (CR → F)	5.17	15	43	PR	36	Discontinue (relapse)
					Cyc → LEF* (CR → F)						
					RTX (NR)						
2	45	F	12	III	Pulse steroids (CR → F)	1.34	5	39	CR	84	Follow-up
					Cyc (PR → F)						
					MMF (NR)						
					TAC (NR)						
3	18	F	6	III + V	MMF (PR → F)	2.33	10	75.5	PR	132	Follow-up
					TAC (PR → F)						
4	49	F	7	III + V	Cyc → MMF* (CR → F)	4.92	0	63	PR	12	Discontinue (SAE)
					TAC (NR)						
5	55	F	18	III + V	CYC (CR → AE)	3.33	10	77	CR	132	Follow-up
					LEF (NR)						
					Cyc (NR)						
					MMF (PR → F)						
					TAC (PR → F)						
6	29	F	6	IV + V	TAC (NR)	3.48	0	35	CR	118	Follow-up
					MMF (PR → F)						
7	30	F	23	IV	Cyc (CR → F)	6.01	15	72	PR	24	Discontinue (relapse)
					MMF (PR → F)						
					Cs (NR)						
					LEF (NR)						
8	31	F	5	III + VI → IV + V^§^	Cyc → MMF* (PR → F)	1.96	10	52	NR	28	NR
					TAC (NR)						
9	32	F	5	III + V	Cyc (NR)	6.01	35	155	CR	36	Discontinue (extra-renal relapse)
					MMF (NR)						
					Sirolimius (NR)						
10	26	F	4	III → V^§^	Cyc (NR)	13.79	0	105	Lost	4	Lost to follow-up
					Cs (NR)						
					TAC (NR)						
11	46	M	6	III	Cyc (PR → F)	6.24	10	72	PR	16	Follow-up
					MMF (PR → F)						
12	19	F	2	IV + V	LEF (NR)	1.32	0	40.2	PR	36	Discontinue (relapse)
					MMF (PR → F)						
13	24	M	10	III + V	Cyc (NR)	2.21	0	81	PR	32	Follow-up
					Cs (NR)						
					MMF (NR)						
					TAC (NR)						
					LEF + *Tripterygium* (NR)						
					Cyc (NR)						
14	34	F	6	IV	Cyc → LEF* (CR → F)	2.57	10	43	CR	36	Follow-up
					Cs (CR → F)						
					MMF (NR)						
					TAC (NR)						
					AZA (NR)						

*TPU* total proteinuria, *Cyc* cyclophosphamide, *AZA* azathioprine, *Cs* cyclosporine, *LEF* leflunomide, *RTX* rituximab, *MMF* mycophenolate mofetil, *TAC* tacrolimus, *CR* complete remission, *PR* partial remission, *NR* no response, *SAE* severe adverse event

*Represents cyclophosphamide (typically 6 months) in sequence with other immunosuppressive agents. In this situation, the patients usually achieved PR or CR when the cyclophosphamide treatment ended

^§^Patients agreed to repeated renal biopsies before iguratimod treatment

At enrollment, the patients’ median age was 30.5 years (interquartile range (IQR) 25.5–45.25 years), and the median amount of proteinuria 3.41 g/24 h (IQR 2.10–6.01 g/24 h). None of the patients had detectable evidence of extra-renal disease, probably because all the patients had received long-term steroid and immunosuppressive therapy. The median prednisone dosage was 10 mg/day (IQR 0–10 mg/day), 13/14 patients receiving prednisone at a dosage of no more than 15 mg/day. The median serum C3 concentration was 0.763 g/L (IQR 0.586–1.021 g/L), and the median anti-dsDNA concentration 23.64 IU/mL according to radioimmunoassay (IQR 18.05–66.00 IU/mL). There were no significant differences in baseline serum C3 or anti-dsDNA concentrations between patients who had and had not discontinued iguratimod treatment. Details of serum C3 and anti-dsDNA concentration are shown in Figure S1 (see Additional file 1).

### Efficacy outcomes

**Fig. 1:**
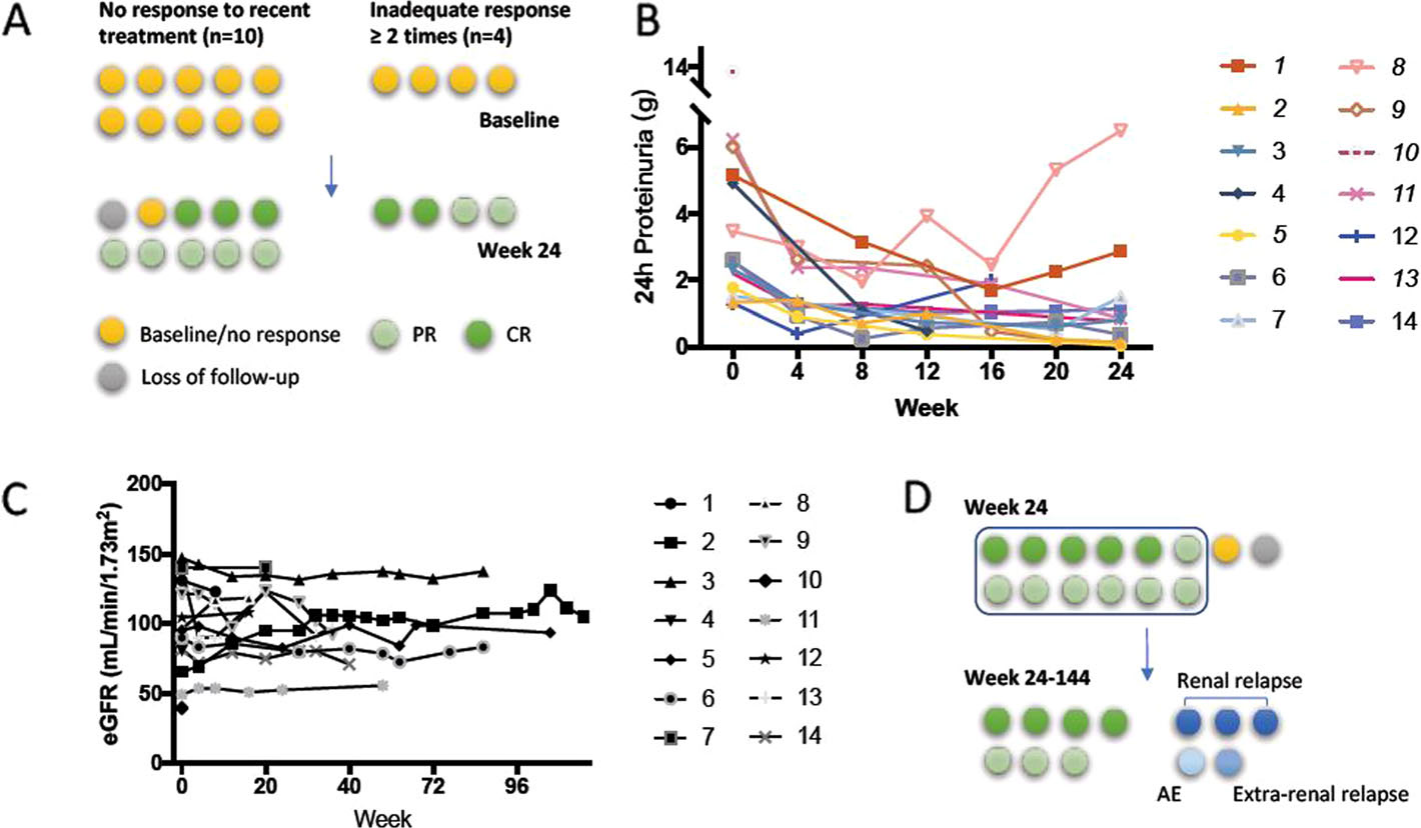
**a** Enrollment and primary outcome of induction treatment at week 24. Each spot represents one patient. **b** Changes of proteinuria during a 24-week follow-up. **c** Estimated GFR (eGFR) during follow-up, calculated by the EPI formula. **d** Outcomes of maintenance treatment for responded patients since week 24. CR, complete remission; PR, partial remission; AE, adverse event

One patient was lost to follow-up after two visits. The other thirteen patients were eligible for evaluating renal response. The renal response rate was 92.3% (12/13) at week 24. Renal CR was achieved by 38.5% (5/13) of patients and PR by 53.8% (7/13). The median duration of response was 12 weeks, with IQR of 4–18 weeks. One patient showed no response after 6 months of iguratimod treatment and therefore was not included in the extended follow-up part of the study (Fig. 1a, b).

The 12 patients who achieved initial response continued attending for follow up. Seven of these patients (four with CR and three with PR) finished up to 144 weeks of follow-up (median follow-up time 80 weeks, IQR 48–80 weeks) with stable amounts of urine protein. Three of the patients (3/12, 25%) developed renal relapse (median flare time 80 weeks) and accordingly discontinued iguratimod. Regardless of remission status, the estimated glomerular filtration rate (eGFR) of all patients was stable, most being over 90 mL/min/1.73 m^2^ (Fig. 1c). Most of our patients had abnormal urine sediment (9/14) at baseline, including hematuria, pyuria, and pathological casts (Table S2, see Additional file 1). Interestingly, patients with baseline active urine sediments tended to be more likely to subsequently achieve CR; however, this tendency was not statistically significant (Table S3, see Additional file 1).

During the extended follow-up period, two patients exited the study for extra-renal reasons: one had thrombocytopenia and worsening of serum anti-dsDNA and C3 after initial CR and required high dose steroids. The other had severe anemia shortly after commencing iguratimod treatment; this was resolved after stopping iguratimod (Fig. 1a).

As to the overall lupus disease activity, only one patient had evidence of extra-renal disease during follow-up, as mentioned above. Yet as a whole, serum anti-dsDNA antibody and C3 concentrations of all patients did not differ significantly between baseline and follow-up (Fig. S1, see Additional file 1).

### Safety profile

During follow-up, most adverse events were mild, such as the common cold and mild decreases in white blood cell counts. The only exception was that one patient developed severe anemia after 12 weeks of treatment. Her hemoglobin was 80 g/L in week 7, yet she insisted on another month of treatment because of a surprising decrease in her proteinuria, which had not decreased in the past 2 years. At week 12, her proteinuria had decreased from 3.92 g/24 h at baseline to 0.47 g/24 h; however, her hemoglobin concentration had fallen from within the normal range at baseline to 32 g/L. A series of tests, including a bone marrow smear, ruled out hemolysis, occult bleeding, and dysplasia. Given that we were unable to identify a clear explanation for her anemia, drug-related reasons had to be considered. We therefore stopped her iguratimod and gave her a transfusion and erythropoietin. Her anemia had been resolved 2 weeks later and remained stable thereafter, despite a rebound of proteinuria.

Impaired liver function has been the most common adverse effect of iguratimod during clinical trials and post-market surveillance [14, 24]. One patient in the present study had a transient increase in alanine aminotransferase concentration that resolved spontaneously within 2 weeks (Fig. S2, see Additional file 1).

## Discussion

CYC, MMF, and rituximab are the agents recommended for the treatment of refractory LN by the EULAR guidelines [4]. However, this regimen does not guarantee a treatment response. In our study, six patients had had inadequate responses to both CYC and MMF before enrollment and another patient had failed on rituximab. Thus, there is a significant unmet need for new agents and strategies for treating refractory LN.

In this study, we showed for the first time the feasibility and potential efficacy of iguratimod in LN management, with a 92.3% response rate at week 24. This response rate is comparable to that reportedly achieved by other therapies that have been investigated for treating refractory LN, including calcineurin inhibitors [9, 10], rituximab [6–8], and stem cell transplantation [25].

Of note, unlike in most other studies, we did not combine iguratimod with other immunosuppressive agents in this study. Moreover, we did not increase the study patients’ steroid dosages. Fortunately, we enrolled 13/14 patients before their steroids had been increased, the one exception being a patient whose prednisone dosage had been increased to 35 mg/day in other hospitals. Therefore, the renal response observed in the study can validly be attributed to the treatment with iguratimod. It also made the results of the study compelling and reliable despite the absence of a control arm.

We found that patients with baseline active urine sediments tended to be more likely to achieve CR later (4/9 in sediment positive patients vs. 1/4 in sediment negative patients), implying that patients with a more active phenotype may be more likely to respond fully to iguratimod treatment. However, the tendency to an association between baseline kidney damage and treatment outcome was not statistically significant, likely because of our small sample size. With only two non-responders, there was not a significant difference in the positive sediment rate between the responders and non-responders.

During the extended follow-up period, three (25%) of the responding patients had renal relapses. None of them had responded to the medication they had received before iguratimod and none of them achieved CR on iguratimod. Given that the achievement of CR is a critical predictor of relapse-free remission, our findings are consistent with those reported by other researchers in similar settings. The flare rate in our study was comparable to that previously reported, namely 12–64%, the rate varying according to race, pathological distribution, and duration of follow-up [26]. Thus, iguratimod could have a role in the maintenance therapy of LN.

Targeting B cell/plasma cells is a promising and attractive strategy for treating refractory LN. Notable B cell depletion by rituximab has been reported [6], and this agent has been recommended despite failing in the initial randomized controlled study [27]. In addition, the proteasome inhibitor bortezomib, which targets plasma cells, has been shown to be effective for treating refractory LN in the short term [28]. These results support the rationale of using iguratimod, which is a B cell terminal differentiation inhibitor, to treat refractory LN.

In addition, iguratimod has been shown to inhibit multiple inflammatory cytokines and chemokines that are involved in LN, such as interleukin (IL)-17, macrophage migration inhibitory factor, IL-6 and IL-1β, and NF-κB activation [18, 29–31]. The suppression of both autoreactive B cells and inflammation suggest that iguratimod may be an effective treatment for LN.

One of the concerns raised by our findings is the severe anemia that one patient developed during treatment (the only serious adverse effect reported). Routine clinical tests, including a bone marrow smear, failed to reveal the exact mechanism of the anemia, probably because of our preemptive use of erythropoietin. In fact, post-market surveillance has shown that anemia is a common AE [24]. If our patient had stopped the treatment promptly, this might not have been a serious adverse effect.

The major limitation of the current study is the small sample size, which was mainly attributable to our stringent inclusion criteria and the study design. We carefully controlled every confounding factor that might have interfered with interpretation of the results. We chose patients with active renal manifestations only, stopped their immunosuppressants to rule out any residual effects from them, and maintained low dose steroids and other treatments to exclude the confounding effect of steroids. We believe the results of the current study clearly show the value of iguratimod in treating LN. On the basis of the current findings, we are performing a randomized controlled clinical trial to compare the efficacy of iguratimod with that of CYC-azathioprine sequential therapy in the induction therapy of active LN (NCT02936375).

Another limitation of this study is that in most patients, renal biopsies were performed at the time of onset of proteinuria. After several treatment regimens and with time, the pathology may have changed, influencing the effects of treatment. A low rate of repeat biopsy is a common problem [32]; only three of our patients agreed to repeat biopsies.

## Conclusion

Our findings suggest that the novel immunomodulatory drug iguratimod may be a new candidate for the treatment of LN. More studies are warranted to verify the efficacy of iguratimod in the treatment of LN as well as other manifestations of SLE.

## Supplementary information

**Additional file 1.** Details of Figure S1, Figure S2, Table S1, Table S2, Table S3 are shown in Additional file 1.

**Additional file 2.** Details of patients’ history before enrollment.

## Data Availability

The datasets used and/or analyzed during the current study are available from the corresponding author on reasonable request.

## Abbreviations

C3: Complement 3
CR: Complete remission
CYC: Cyclophosphamide
dsDNA: Anti-double strand DNA
eGFR: Estimated glomerular filtration rate
LN: Lupus nephritis
MMF: Mycophenolate mofetil
PR: Partial remission
SLE: Systemic lupus erythematosus
