# Supplementary material for: Iguratimod as an alternative induction therapy for refractory lupus nephritis: a preliminary investigational study
Source: Arthritis Res Ther. 2020 Mar 30;22:65. doi: 10.1186/s13075-020-02154-7 (PMC7106733; doi:10.1186/s13075-020-02154-7)
Supplement: Supplementary file 1 — Additional file 1. Details of Figure S1, Figure S2, Table S1, Table S2, Table S3 are shown in Additional file 1. [file 13075_2020_2154_MOESM1_ESM.docx]

**Supplementary figures and tables**

**Iguratimod as an Alternative Induction Therapy for Refractory Lupus Nephritis: A Preliminary Investigational Study**

Yuening Kang*, Qingran Yan* #, Qiong Fu*, Ran Wang, Min Dai, Fang Du, Qing Dai, Ping Ye, Chunmei Wu, Liangjing Lu# and Chunde Bao#


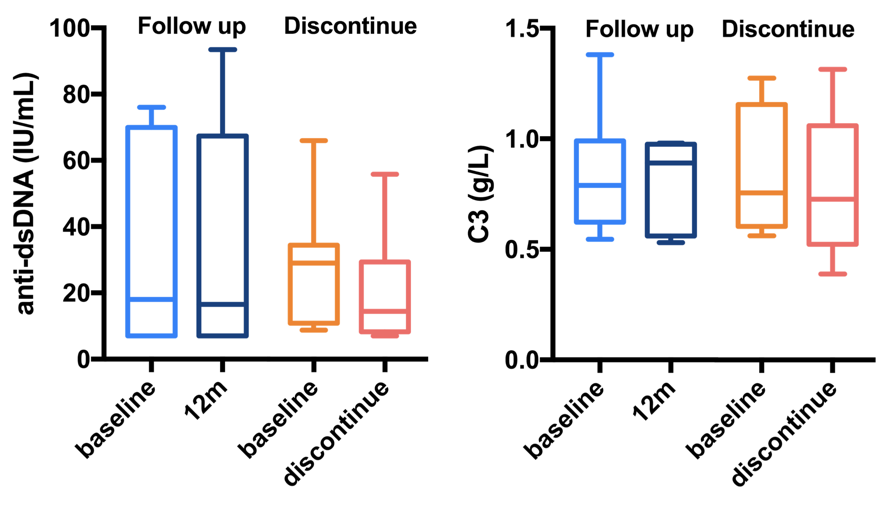


Fig S1. Serum anti-dsDNA and C3 levels during follow-up. The boxes represent the medians and quartiles, and the bars represent the maximum and minimum numbers. No statistical significance between baseline anti-dsDNA or C3 levels of follow-up patients and withdrawn patients was observed, and no statistical significance was observed between the paired baseline and follow-up data (Mann-Whitney test).

Fig S2. Serum alanine aminotransferase (ALT) of each patient during the follow-up. One patient had transient elevated ALT and recovered spontaneously.

| patient | ACEI/ARB | (dose/d) |
| --- | --- | --- |
| 1 | valsartan | 80mg |
| 2 | none |  |
| 3 | none |  |
| 4 | none |  |
| 5 | valsartan | 80mg |
| 6 | none |  |
| 7 | none |  |
| 8 | valsartan | 80mg |
| 9 | valsartan | 80mg |
| 10 | none |  |
| 11 | Losartan | 100mg |
| 12 | none |  |
| 13 | Benazepril | 10mg |
| 14 | none |  |

Table S1. The accompanying use of angiotensin converting enzyme/receptor inhibitor (ACEI/ARB) for each patient. None of them had dose adjustment once got enrolled.

| Patient | Microscopic red blood cells (0-4/HP) | Microscopic leukocyte (0-5/HP) | Pathological casts (0.0-1.0/μl) | Abnormal urine sediment (+/-) |
| --- | --- | --- | --- | --- |
| 1 | 11.7 | 73.5 | 0 | + |
| 2 | 6.9 | 8.6 | 0 | + |
| 3 | 27 | 20 | 6 | + |
| 4 | 2 | 0 | 0 | - |
| 5 | 3.6 | 0 | 0 | - |
| 6 | 2.5 | 56 | 0 | + |
| 7 | 3.2 | 1.1 | 0.1 | - |
| 8 | 43 | 36 | 1 | + |
| 9 | 113 | 13 | 1 | + |
| 10 | 2.1 | 0 | 0 | - |
| 11 | 164 | 137 | 2 | + |
| 12 | 3.6 | 1.2 | 0 | - |
| 13 | 6.5 | 2.3 | 0 | + |
| 14 | 8.9 | 0 | 0 | + |

Table S2. Urine sediments of each patient at baseline.

|  | CR | Non-CR |
| --- | --- | --- |
| Abnormal sediment* | 4 | 5 |
| Normal sediment | 1 | 3 |
|  | Responder† | Non-responder |
| Abnormal sediment | 8 | 1 |
| Normal sediment | 4 | 0 |

Table S3. Analysis of baseline urine sediments and treatment outcomes. * Abnormal sediment refers to hematuria, pyuria or pathological casts. †Responder refers to patients achieving CR or PR. The data were tested by Fisher’s exact test and no statistical significance were found.
