# Supplementary material for: Iguratimod as an alternative induction therapy for refractory lupus nephritis: a preliminary investigational study
Source: Arthritis Res Ther. 2020 Mar 30;22:65. doi: 10.1186/s13075-020-02154-7 (PMC7106733; doi:10.1186/s13075-020-02154-7)
Supplement: Supplementary file 2 — Additional file 2. Details of patients’ history before enrollment. [file 13075_2020_2154_MOESM2_ESM.docx]

The ages mentioned are the patients’ ages at entry to the study.

Patient 1

This 30-year-old woman was diagnosed with LN (Class IV) in 2006. She received monthly CYC plus high dose steroids for four months and achieved CR; however, she was then lost to follow-up and her disease flared up. She accordingly restarted CYC treatment, again achieving CR. She was then commenced on cyclosporine. However, her disease relapsed soon after reducing her steroid dosage. She then received a third round of pulse CYC plus high dose steroids, again achieving remission. This time leflunomide was selected as maintenance treatment. She continued this for 1 year and then relapsed again. She was then treated with two 500 mg rituximab injections at 2-week intervals but showed no improvement in proteinuria during another 6-month follow-up. She agreed to a second kidney biopsy before commencing iguratimod treatment. Examination of the biopsy material resulted in a diagnosis of Class III LN.

Patient 2

This 45-year-old woman was diagnosed with LN (Class III) in 2007. She initially received only high dose steroids and achieved CR. She stopped this therapy without her physician’s permission because she was concerned about adverse effects and had a renal relapse. She was persuaded to receive monthly CYC plus high dose steroids in 2010 and achieved CR. Her pulse CYC was reduced to every 3 months and she had another relapse in 2012. Considering her cumulative dose of CYC, she was then given MMF with high dose steroids; however, her proteinuria had decreased by less than 50% after 9 months of this treatment. Tacrolimus was tried in 2014 but did not achieve a response.

Patient 3

This 18-year-old woman was diagnosed with LN (Class III+V) in 2013. Considering that she was young and single, she was treated with MMF plus steroids. She achieved PR but relapsed 1 year later, at which stage she received tacrolimus with a higher steroid dosage. She again achieved a PR again but had a renal relapse 18 months later.

Patient 4

This 49-year-old woman was diagnosed with LN (Class IV+V) in 2012. She received monthly CYC plus high dose steroids, achieved CR, and received leflunomide as maintenance treatment. She had a renal relapse 1 year later for which she refused another round of CYC because she was concerned about induction of premature menopause. MMF was substituted for the leflunomide and the steroid dosage was increased for 6 months; however, no response was achieved. She then tried tacrolimus for another 6 months with no improvement in her proteinuria.

Patient 5

This 55-year-old woman was diagnosed with LN (Class III+V) in 2001. She initially received monthly CYC and achieved CR. She stopped CYC because of hair loss and premature menopause 5 months later. She then took only steroids with hydroxychloroquine and her disease was stable until she had a renal flare in 2011. She then received leflunomide plus steroids for 6 months yet achieved no response. Accordingly, she was then persuaded to accept another round of monthly CYC plus high dose steroids; however, no response was achieved 4 four months of this combination. She received MMF plus high dose steroids and finally achieved a PR. Another renal flare occurred 2 years after this PR, for which she received tacrolimus and again achieved PR. Unfortunately, she had another renal flare in 2017.

Patient 6

This 29-year-old woman was diagnosed with LN (Class III+V) at the end of 2013, for which she received high dose steroids plus only two times of monthly CYC. Because of concern about the toxic effects of cyclophosphamide on the reproductive system, she was started on tacrolimus (2 mg daily) plus steroids in 2014. Four months later, no response had been achieved. She therefore received MMF (1.5 g daily) and achieved PR. One year later, she had a renal relapse.

Patient 7

This 30-year-old woman was diagnosed with LN (Class IV) in 1996. She initially received monthly CYC (0.8 g/m^2^ monthly) plus high dose steroids. Once CR had been achieved, the frequency of CYC was gradually reduced and it was stopped 2 years later. Her disease remained stable for years with low dose steroids and hydroxychloroquine. In 2007, she experienced her first renal flare and began MMF (2.0 g daily). She achieved CR and the MMF dose was reduced slowly to discontinuation. She had a second flare in 2014 during a pregnancy, for which her steroid dosage was increased. She restarted MMF after delivery. This time only a PR was achieved and her disease flared up again 1 year later. She then received cyclosporine for approximately 10 months but showed no response. She was subsequently treated with leflunomide, still with no response.

Patient 8

This 31-year-old woman was diagnosed with LN (Class III+V) in 2014. She initially received CYC (0.8 g/m^2^ monthly) plus high dose steroids. She achieved PR and began MMF maintenance treatment. This did not result in a CR and her disease flared up again in 2016. Tacrolimus with increased steroids was substituted for MMF; however, she showed no response in 7 months. Before commencing iguratimod, she agreed for a second renal biopsy, which resulted in a pathological diagnosis of LN Class IV+V.

Patient 9

This 32-year-old woman was diagnosed with LN (Class III+V) in 2014. She was initially treated with CYC (0.8 g/m^2^ monthly) plus high dose steroids but showed no response by the end of sixth month. She received MMF for 8 months but again showed no response. Next, sirolimus was tried for 9 months, again with no evidence of remission.

Patient 10

This 26-year-old woman was diagnosed with LN (Class III) in 2015. She initially received CYC (0.5 g/m^2^ monthly) plus high dose steroids. She had no response by the end of sixth month and accordingly received cyclosporine treatment for another 6 months, again with no response. She was then treated with tacrolimus (1.5 mg daily) plus high dose steroids. Despite some decrease in proteinuria, she did not achieve remission during 9 months of treatment. Before she entering the present study, she underwent second renal biopsy, which showed Class V LN.

Patient 11

This 46-year-old man was diagnosed with LN (Class III) in 2013. He was treated with monthly CYC (0.8 g/m^2^ monthly) plus high dose steroids and achieved PR 7 months later. The frequency of CYC was then gradually reduced to every 3 months. The patient never achieved CR with CYC treatment. He had a renal relapse in 2015 and started MMF treatment. Though his proteinuria decreased, he did not achieve CR and relapsed in 2017.

Patient 12

This 19-year-old woman was diagnosed with LN (Class IV+V) in 2017. She was initially treated with leflunomide for 6 months; however, this was ineffective. She was then treated with MMF and achieved PR; however, 6 months later she had a renal relapse.

Patient 13

This 24-year-old man was diagnosed with LN (Class III+V) in 2009. He received CYC (0.8 g/m^2^ monthly) plus high dose steroids but showed no improvement in proteinuria after 6 months of this therapy. He later received multiple therapies, including cyclosporine, MMF, tacrolimus, and leflunomide combined with tripterygium. She received each of these drugs for at least 6 months, yet showed no response to any of them. Before enrollment in the present study, the patient was persuaded to undergo another round of pulse cyclophosphamide with high dose steroids. Unfortunately, he failed to show a response with 5 months of this therapy.

Patient 14

This 34-year-old woman was diagnosed with LN (Class IV) in 2013. She received pulse cyclophosphamide (0.8 g/m^2^ monthly) plus high dose steroids and achieved complete remission. She then received leflunomide (20 mg daily) as maintenance treatment. She had a renal relapse 6 months after remission and cyclosporine was substituted for the leflunomide. During this treatment, she was found to have a left renal chromophobe cell carcinoma and stopped treatment to undergo surgery. She restarted treatment with cyclosporine plus steroids in September 2014 and achieved CR, which was maintained for approximately 2 years. She had another renal relapse in 2016 and started MMF treatment. Unfortunately, this time she showed no response. Azathioprine and tacrolimus were substituted for MMF, each for 6 months, but she failed to respond to either of these drugs.
